# Mitomycin C enhanced the efficacy of PD-L1 blockade in non-small cell lung cancer

**DOI:** 10.1038/s41392-020-0200-4

**Published:** 2020-08-28

**Authors:** Min Luo, Fang Wang, Hong Zhang, Kenneth K. W. To, Shaocong Wu, Zhen Chen, Shaobo Liang, Liwu Fu

**Affiliations:** 1 State Key Laboratory of Oncology in South China; Collaborative Innovation Center for Cancer Medicine; Guangdong Esophageal Cancer Institute, Sun Yat-Sen University Cancer Center, 510060 Guangzhou, China; 2 School of Pharmacy, Faculty of Medicine, the Chinese University of Hong Kong, Hong Kong, China

**Keywords:** Immunotherapy, Cancer therapy

## Abstract

Programmed death ligand 1 (PD-L1) immune checkpoint inhibitors are promising therapeutic agents for treating cancers but the response rate is <20%. Some chemotherapeutic drugs could also activate an anticancer immune response to kill cancer cells, apart from their direct cytotoxicity. Our study investigated the combination of chemotherapeutic drugs with PD-L1 antibody to enhance the response rate of PD-L1 blockade. Non-small cell lung cancer (NSCLC) cells were pre-treated with mitomycin C (MMC) and then co-cultured with peripheral blood mononuclear cells (PBMCs) to investigate the effect of the combination of MMC with PD-L1 antibody. The drug combination was also evaluated in vivo in Lewis lung cancer (LLC) cells-bearing C57BL/6 mice. MMC increased the expressions of PD-L1 and MHC-I in NSCLC cells in vitro and in vivo and enhanced the cytotoxic effect of lymphocytes on NSCLC in vitro. In LLC-bearing mouse model, the combination of MMC and PD-L1 antibody was found to be more effective in retarding tumor growth and prolonging overall survival than either single treatment alone, which was associated with increased lymphocyte infiltration and granzyme B release. Mechanistically, MMC activated the ERK pathway, which subsequently enhanced the binding of c-JUN to the PD-L1 promoter and recruited its co-factor STAT3 to increase PD-L1 expression. The upregulated ERK pathway was shown to activate p65 to increase the MHC-I expression. MMC was shown to enhance the efficacy of PD-L1 blockade in NSCLC cells. Further study is warranted to translate the findings to clinical application.

## Introduction

Human body makes use of T cells to selectively recognize and kill external pathogens and unhealthy cells, including cancer cells, by coordinating innate and adaptive immune responses. The programmed cell death-1 (PD-1) receptor is a key inhibitory immune checkpoint protein expressing on the surface of activated T cells. Its ligand programmed death ligand 1 (PD-L1), also known as B7-H1^1^ is commonly expressed in many cell types, including T cells, B cells, monocytes, antigen process cells (APCs), and epithelial cells.^2,3^ The PD-1/PD-L1 interaction limits the development of T cells response, thereby ensuring the activation of immune system appropriately.^2,3^ Cancer cells can exploit various immune checkpoints to evade immune detection and elimination. Overexpression of PD-L1 has been observed in a variety of solid tumors or on non-transformed cells in the tumor microenvironment.^4^ The interaction of PD-L1 on the surface of tumor cells and the PD-1 receptors on activated T cells leads to inhibition of cytotoxic T cells. Consistently, the high expression of PD-L1 in tumors is correlated with poor clinical prognosis in cancer patients.^5–7^ Along with the recent development of cancer immunotherapy, blockade of PD-L1/PD-1 interaction or down-regulation of PD-L1 expression in cancer cells has been reported to enhance antitumor immunity activity and to inhibit tumor growth.^8,9^ A few immunotherapeutic agents against PD-1/PD-L1, including nivolumab, pembrolizumab, durvalumab, atezolizumab, and avelumab, have been recently approved by the US Food and Drug Administration, which have revolutionized the treatment of a subset of cancer patients, including non-small cell lung cancer (NSCLC) with high expression of PD-L1. While durable tumor regression could be achieved in some patients, only <20% of patients respond to these immunotherapeutic agents.^10^

Various classical chemotherapeutic drugs are known to alter the tumor microenvironment to activate immune response, apart from their well-established direct cytotoxic effect on cancer cells.^11^ Cyclophosphamide has been reported to deplete regulatory T (T_Reg_) cells in preclinical adoptive T cells and vaccine models,^12^ which may augment immunotherapies in patients. A few other cytotoxic chemotherapeutic drugs, including 5-fluorouracil, gemcitabine, and taxanes have been reported to cause a decrease in myeloid-derived suppressor cells (MDSCs).^13,14^ On the other hand, some chemotherapeutic regimens have been shown to alter the immune system to potentiate the anticancer response to immune checkpoint blockade. Histone deacetylase inhibitor are found to synergize with CTLA-4 or PD-1 blockers to eradicate primary tumor and metastases in murine models.^15^ Moreover, neoadjuvant chemotherapy has been demonstrated to stimulate tumor-infiltrating lymphocytes (TILs) and upregulate PD-L1 expression in tumor cells in cancer patients.^16^ Therefore, chemotherapeutic drugs exhibiting these immunostimulatory properties represent attractive candidates for combination with immunotherapy. It is highly desirable to identify specific anticancer drugs that can increase the immunogenicity of cancer cells and subsequently expand the benefit of anti-PD-L1 treatment.

In this study, we evaluated the effect of serveral conventional chemotherapeutic drugs on PD-L1 expression in NSCLC cells. Mitomycin (MMC) was found to synergize with PD-L1 antibody in NSCLC cells in vitro and in vivo. Compared with the individual single agents, the combination therapy (MMC + PD-L1 antibody) was found to be more effective in increasing the number of tumor-infiltrating lymphocytes (TILs) and promoting the secretion of the cytotoxic serum protease (granzyme B) from T cells to induce apoptosis of targeted cancer cells. Moreover, the combination treatment was also found to elevate MHC-I expression in NSCLC cells. The in vitro findings were also substantiated by the remarkable increase in TILs and elevated levels of PD-L1 and MHC-I in tumor specimens from patients after MMC therapy. Mechanistically, MMC was shown to activate the ERK pathway, which subsequently facilitated the binding of c-JUN to the PD-L1 promoter and recruited its co-factor STAT3 to upregulate PD-L1 expression. Moreover, the upregulated ERK pathway was also shown to activate p65 to increase MHC-I expression. In summary, this is the first study systematically evaluated the potentiation of antitumor effect from PD-L1 checkpoint inhibitors by a classical chemotherapeutic drug MMC in NSCLC.

## Materials and methods

### Cell lines

The human NSCLC cell lines (H460, A549, and H1299), LLC cells, normal cell lines (HUVEC and NCM460) and 293T cells were obtained from the American Type Culture Collection (ATCC, Manassas, VA, USA). The identity of the cell lines were validated by short-tandem-repeat (STR) (except for LLC). The human cancer cells were cultured in RPMI supplemented with 10% fetal bovine serum at 37 °C in a humidified 5% CO_2_ incubator. 293T and LLC were cultured in DMEM supplemented with 10% fetal bovine serum at 37 °C in a humidified 5% CO_2_ incubator. Primary T cells were grown in T cell medium (RPMI-1640 supplemented with 10% human serum, 5% l-glutamine-penicillin–streptomycin solution (Sigma-Aldrich,USA) and IL-2, 100 IU per mL.

### Chemotherapeutic drugs and antibodies

The following reagents were used: MMC, nifuroxazide, U0126, SP600125, and cycloheximide were purchased from Selleck Chemicals (Houston, TX, USA). Stock solutions of these reagents were prepared in dimethyl sulfoxide (DMSO) or water at a concentration of 5 ~10 mmol/L and they were stored at −20 °C before use. Working solutions of these reagents were diluted from the stock with cell culture medium and they were used at concentrations ranging from 0.5 μmol/L to 20 μmol/L for the treatment of cell lines. The following antibodies were purchased from Cell Signaling Technology (Danvers, MA, USA): MHC-I, p65, phospho-p65, phospho-STAT3, STAT3, c-JUN, phospho-c-JUN, ERK, phospho-ERK, JNK, and phospho-JNK. Human B7H1/PD-L1 polyclonal was purchased from Santa Cruz Biotechnology (Dallas, TX, USA); and IFNGR1 and GAPDH were from Abcam (Cambridge, UK). Polyclonal goat anti-mouse and goat anti-rabbit secondary antibodies were obtained from R&D Systems (Minneapolis, MN, USA).

### MTT assay

The drugs cytotoxicity tests in vitro were evaluated using the MTT colorimetric assay as described previously.^17^ Briefly, cells were seeded in 96-well plates at appropriate density, incubating for 24 h at 37 °C. The toxicity of MMC indicated that more than 80% of cells survived at 0.4 μmol/L, so we used MMC 0.4 μmol/L for the subsequent experiments. The 20 μL of MTT solution (5 mg/mL) was added to each well after 68 h of incubation. The MTT-medium was discarded and the resulting formazan crystals were dissolved with DMSO. The cytotoxicity was measured by a Model 550 Microplate Reader (Bio-Rad, Hercules, CA, USA). All experiments were repeated at least three times, and the mean value±SD was calculated.

### Reverse transcription and quantitative real-time PCR

Reverse transcription and quantitative PCR were performed as previously described.^18^ The primers were listed below: c-JUN, F: CAGGTGGCACAGCTTAAACA, R: CGCAACCAGTCAAGTTCTCA; IFNGR1, F: CATCACGTCATACCAGCCATTT, R: CTGGATTGTCTTCGGTATGCAT; MHCI, F: AGTGGGCTACGTGGACGACA, R: ATGTAATCCTTGCCGTCGTA; p65, F: ATCAATGGCTACACAGGA, R: CTGTGGATGCAGCGGTCC; STAT3, F: CTTGACACACGGTACCTGGA, R: CTTGCAGGAAGCGGCTATAC; PD-L1, F: TATGGTGGTGCCGACTACAA, R: TGCTTGTCCAGATGACTTCG; β-actin, F: TCCTGTGGCATCCACGAAACT, R: GAAGCATTTGCGGTGGACGAT. Data were normalized to β-actin levels in the samples in triplicates. Relative expression versus β-actin expression is calculated using the ΔCt method using the following equations: ΔCt (Sample) = Ct (Target) − Ct (Reference); relative quantity = 2^−ΔΔCt^.

### Protein extraction and western blot analysis

Western blot analysis was performed as described previously.^19,20^ Briefly, the whole cell lysates were prepared by using the RIPA lysis buffer (Santa Cruz Biotechnology, Heidelberg, Germany). The protein samples were subjected to SDS-PAGE (10% Tris/Glycine gel) and the separated proteins were transferred onto a polyvinylidene difluoride membrane (Millipore, Danvers, MA, USA) for immunoblotting. Membranes were probed with primary antibodies for the respective target proteins, followed by washing and further incubation with secondary antibody conjugated with horseradish peroxidase (Amersham GE Healthcare, Pittsburgh, PA, USA). Bands were visualized by using a chemiluminescent reagent (Pierce ECL kit, Thermo Fisher Scientific, USA).

### Luciferase assays

Luciferase assays were performed as described in our previous reports.^21,22^ The MHC-I promoter was purchased from GeneCopoeia Inc (Rockville, MD, USA). The 868-bp PD-L1 promoter fragment (https://www.ncbi.nlm.nih.gov/gene/29126) was PCR-amplified from H460 cells genomic DNA and inserted into the promoter-less plasmid pGL3-Basic (Promega, USA) designated as p868. A series of 5′-deletions were produced by PCR using p868 as a template with the different 5′ primers (F) and a common 3′ primer (R) as follows: p-868, F: TGAACCTAACAGCAGGGAAAAC; p-693, F: AGCCCTGTTTAAGTGTTCTCTG; p-516, F: CAGAGTGGTGGTACGAAAAGAG; p-360, F: GCTTCTAAAGGGTACACTGGAG; R: TTATCATTTTCTAGGCTGGGTG. The products were cloned into pGL3-Basic to generate p693, p516, and p360. The promoter sequences were then interrogated for transcription factor binding sites and transcription factor modules with the use of PROMO (http://alggen.lsi.upc.es/) and the JASPAR database (http://jaspar.genereg.net/). The c-JUN, STAT4, and FOXP3 cDNA vectors were gifts from Professor Jiahuai Han (Xiamen University, China). The relative luciferase activity was examined by Dual Luciferase Assay Kit (Promega, Madison, WI, USA) in accordance with the manufacturer’s protocols.

### Chromatin immunoprecipitation assay

The Chromatin immunoprecipitation (ChIP) assay was performed as described previously.^23^ The SimpleChIP enzymatic chromatin IP kit (Cell Signaling Technology, Beverly, MA, USA) was used. Specific antibody against endogenous c-JUN (Cell Signaling Technology, #9165, USA) was used to immunoprecipitate protein complexes containing c-JUN. Primer sequences specific for a promoter region on PD-L1 for ChIP assay in this study were described: F: CCTAACAGCAGGGAAAACGA, R: ATTTTGGGGAGAGATGGGGT.

### Establishment of knockdown cells and stable PD-L1 knockout cells

Establishment of stable cell lines were performed as previously described.^21,24^ The shRNA vectors for c-JUN, STAT3, IFNGR1, or p65 shRNAs and a no template control (NTC) vector (Dharmacon Inc, Lafayette, CO, USA) were transiently transfected with a pSIH-H1-puro Lentivector Packaging Kit (System Biosciences, Palo Alto, CA, USA). NTC: CAACAAGATGAAGAGCACCAA; c-JUN, sh1: TAGTACTCCTTAAGAACACAA, sh4: GCAGCAGCAGCCGCCGCACCA; STAT3, sh2: CAGGCTGGTAATTTATATAAT, sh3: GGCGTCCAGTTCACTACTA; IFNGR1, sh1: GCCTACACCAACTAATGTTAC, sh4: GACGAGCAGGAAGTCGATTAT; p65, sh1: GCAGATCAGCCAGGCCTCGGC; and p65 sh2: GCTCCAGCCATGGTATCAGCT. The sgRNA oligonucleotides (ACCCCAAGGCCGAAGTCATC) of PD-L1 (SigmaAldrich, USA) were phosphorylated, annealed and cloned into lentiviral expression vectors, lentiCRISPR v2 (Addgene #52961, deposited by Feng Zhang). Transfections were carried out in 6 cm dishes at ∼80% confluency of 293T using Lipofectamine 2000 transfection reagent (Life Technologies, USA) and following the manufacturer’s instructions. H460, H1299, and A549 cells were infected and incubated with the viral particles overnight at 37 °C. At 48 h after transfection, cells were placed under puromycin selection (3 μg/ml for H460, 4 μg/ml for H1299 and A549). Individual clones were verified by western blot and RT-PCR.

### Lactate dehydrogenase assay

Human peripheral blood mononuclear cells (PBMCs) were purified from the blood of healthy volunteers by Ficoll gradient centrifugation and they were used as effector cells in the co-culture system for lactate dehydrogenase (LDH) assay (Solarbio, Beijing). Activated T cells were prepared from PBMCs as previously described.^25^ The 96-well plates were coated overnight with 5 μg/ml anti-CD3 (BD Bioscience, San Jose, CA, USA) and washed twice with PBS before the addition of cells for experiments. PBMCs were plated at 1 × 10^5^ cells per well in complete TCCM medium (IMDM with human AB serum (5%), penicillin–streptomycin, HEPES, 2-mercaptoethanol, and gentamicin). The H460 cells were used as target cells, with or without 24-h pre-treatment by MMC (0.4 μmol/L). H460 cells were co-cultured with the activated PBMCs cells at several effector-to-target (E: T) ratios. The amount of LDH in the supernatant of the co-culture system was detected with the CytoTox96 nonradioactive assay (Promega, USA) according to the manufacturer’s instructions. Cytotoxicity for each E: T ratio was calculated as previously described.^26^

### T cells mediated tumor killing

T-cell-mediated tumor cell killing assay was performed as described previously.^21^ Tumor cells were pre-treated with MMC (0.4 μM) for 24 h. PBMCs were plated at a density of 1 × 10^6^/well in 6-well plates and then co-cultured with tumors cells at 4:1 ratio for 24 h. Anti-human PD-L1 antibody, atezolizumab (Selleck Chemicals, USA) (50 μg/ml) was added afterwards. The co-culture system was maintained for 5 days and the wells were washed with PBS twice to remove PBMCs. The adherent and surviving cancer cells were fixed and stained with crystal violet solution. Cancer cell viability was then estimated by colorimetric determination.

### Flow cytometric analysis

TNF-α and granzyme B analysis were determined by flow cytometric assays performed as previously described.^27^ PBMCs were harvested after co-culture with cancer cells and then treated with brefeldin A (Biolegend, USA) at 37 °C for 3 h before collection for flow cytometric analysis of intracellular granzyme B and TNF-α. Flowjo (Treestar, USA) software was used for the analysis of flow cytometry data. Normalized fluorescence intensities were calculated by dividing the median fluorescence intensities of specific antibodies by the median fluorescence intensities of isotype controls. Results were presented as mean ± SD of three independent experiments.

### Animals

The animal treatment protocol was performed according to previous report.^25^ Female C57BL/6 mice (8–12 weeks old) were purchased from Guangdong Medical Laboratory Animal Center, China. Tumor-bearing mice were established by subcutaneous injection of 2 × 10^5^ LLC cells suspended in 200 μL growth medium into the right flank of immune-competent C57BL/6. The tumor volume and body weight were measured every 3 days. The tumor volume was calculated by the formula: 1/2(length × width^2^). Mice were treated with MMC or anti-mouse PD-L1 antibody (αPD-L1, clone 10 F.9G2; Biolegend, USA) alone, the combination of MMC and PD-L1 antibody, or saline+IgG2bκ (isotope control antibody, clone RTK4530; Biolegend, USA) by intraperitoneal injection (*n* = 6 in each group). MMC (3 mg/kg) or saline was administered intraperitoneally from day 13, every 4 days, after tumor implantation. The PD-L1 antibody therapy 10 mg/kg was administered intraperitoneally weekly on days 16, 23, 30, and 37. When the tumor volume reached ~2000 mm^3^, the mice were defined death and killed.^28,29^ The mice survival analysis was performed by the Kaplan–Meier curves and log-rank test.

### Immunohistochemistry

Immunohistochemical staining and counting of immune cells were performed as previously described.^16,30,31^ The paraffin-embedded tissues sections (3 μm thick) were incubated with primary antibody CD3 (dilution 1:100), CD8 (dilution 1:200), or granzyme B (dilution 1:200), PD-L1 (dilution 1:100), (Cell signaling technology, USA), MHC-I (dilution 1:100) (Proteintech, USA) and the slides were incubated 37 °C for 1 h and HRP-labeled streptavidin solution for 10 min, then stained by diaminobenzidine (DAB).

### Statistical analysis

Statistical analysis was performed by SPSS 19.0 (IBM, SPSS, Chicago, IL, USA) and Graphpad Prism 5.0 (Graphpad Software Inc., CA, USA). All experiments were repeated in triplicate. Data were expressed as mean ± SD. The differences between groups were assessed by Student’s *t*-test or one-way ANOVA. Statistical significance was defined as *p* < 0.05.

## Results

### PD-L1 expression was upregulated by MMC in concentration- and time-dependent manners

**Fig. 1:**
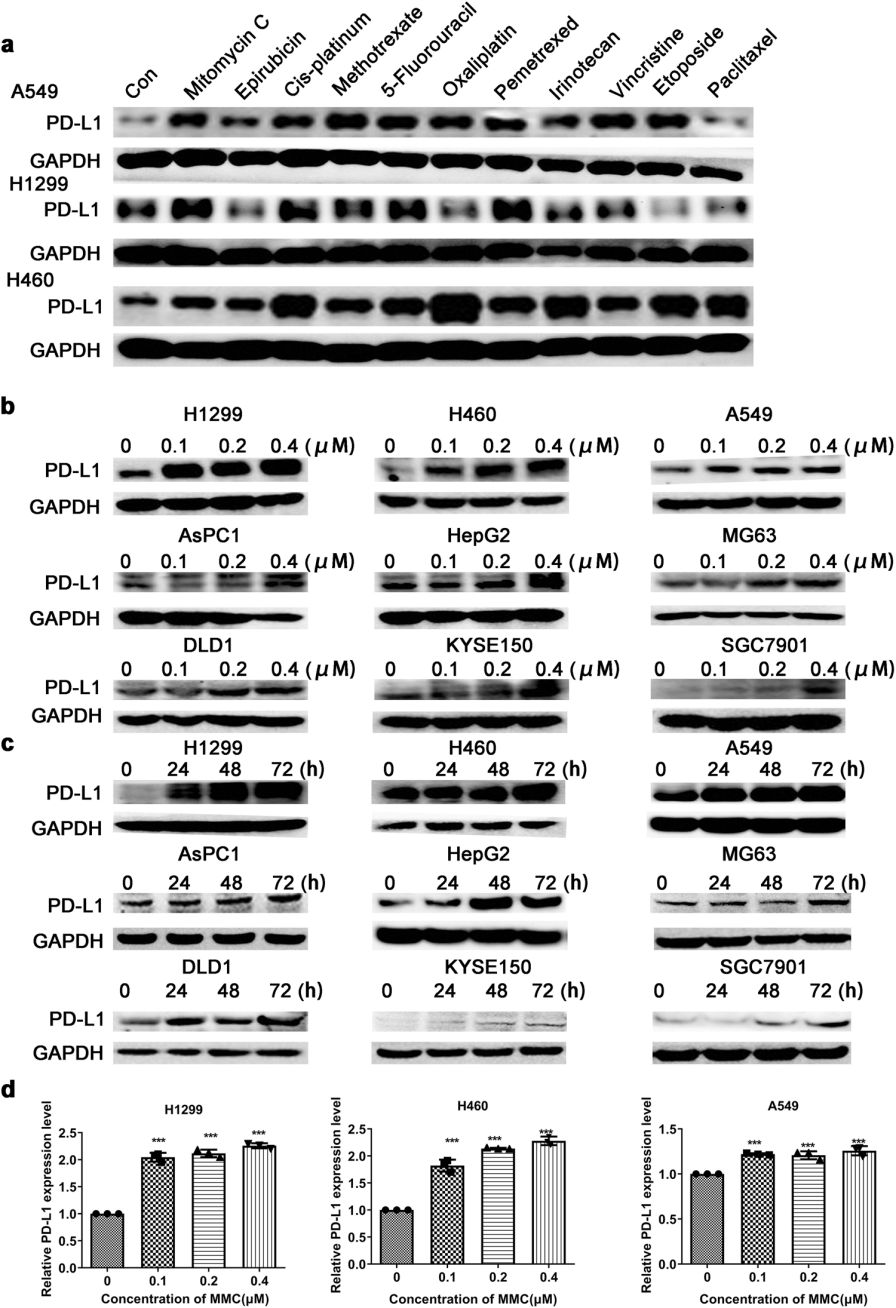
Upregulation of PD-L1 expression in tumor cells following MMC treatment. **a** Western blot analysis of PD-L1 protein expression in three different NSCLC cells (A549, H1299, and H460) after 48-h treatment with different chemotherapeutic drugs. **b**, **c** Representative images showing the western blot analysis of PD-L1 protein expression of the indicated NSCLC cells after treatment with MMC at different concentrations and for different duration. **d** Relative PD-L1 protein expression (normalized with GAPDH) in NSCLC cells after 72-h treatment of MMC (0–0.4 μM). Results are presented as mean ± SD. ***p < 0.001

The effect of several conventional chemotherapeutical drugs at non-cytotoxic concentration on PD-L1 expression in three NSCLC cells (A549, H1299, and H460) was evaluated after 48-h treatment (Fig. 1a). MMC was found to consistently increase PD-L1 expression in all three NSCLC cells (Fig. 1a). To verify whether the upregulation of PD-L1 by MMC is a generalized phenomenon, MMC was evaluated at different concentrations and treatment duration in more cancer cells from different cancer types. The MTT assay was presented in Supplementary Fig. 1. In the NSCLC and all other cancer cells tested, MMC was found to remarkably upregulate PD-L1 expression in concentration- and time-dependent manners (Fig. 1b–d).

### MMC potentiated the anticancer efficacy of PD-L1 blockade in NSCLC in vivo

**Fig. 2:**
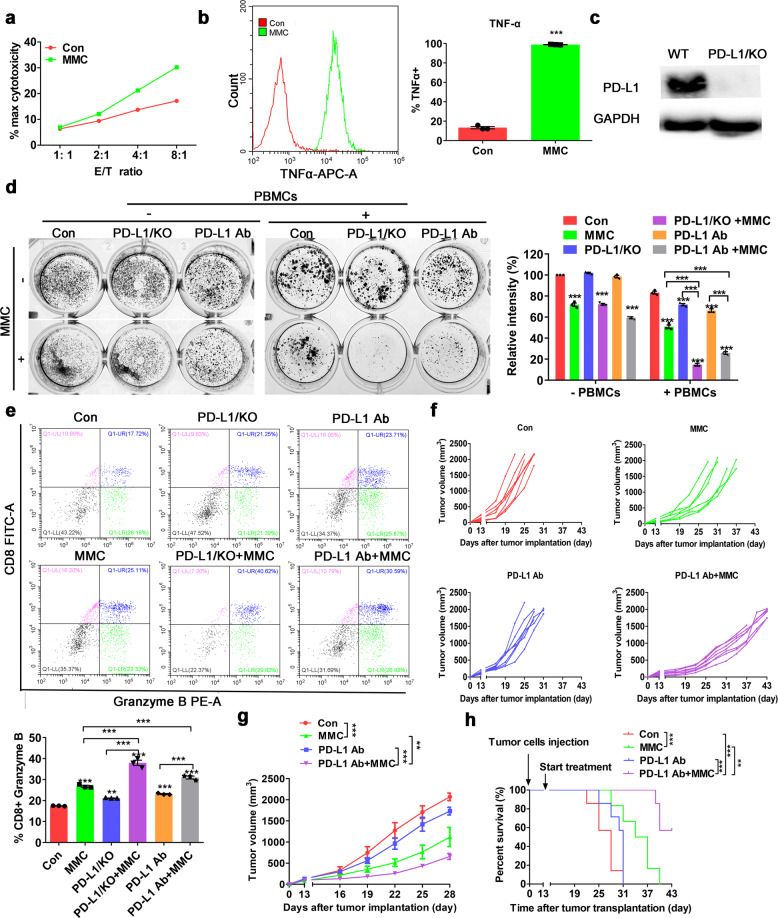
Combination of PD-L1 blockade and MMC produced a synergistic antitumor effect. **a** LDH assay in H460 cells pretreated with MMC (0.4 μM, 24 h). Effector cell (E): PBMC; Target cell (T): H460. **b** Intracellular cytokine staining of TNF-α for CD3^+^ T cells population after incubation with MMC-pretreated H460 cells (0.4 μM, 24 h) for 12 h. The left histogram shows the data from a representative experiment. Means ± S.D. presented on the right bar graph summaries the results from triplicate experiments. **c** The PD-L1 protein expression is essentially knocked out in PD-L1/KO H460 cells. **d** T cell-mediated tumor cell killing assay in PD-L1/KO cells and wild-type control cells with or without MMC pretreatment. Activated T cells and cancer cells were co-cultured in 24-well plates for 4 days and then surviving tumor cells were visualized by Giemsa staining. Relative fold ratios of control cells were summarized in the bar graph. **e** Intracellular cytokine staining of granzyme B of CD8^+^ T cells populations after incubation with H460 cells (with or without MMC-pretreatment). Results were presented in the bar graph as means ± S.D. from triplicate experiments. **f**, **g** The tumor volume curves of LLC-tumor bearing C57BL/6 mice in the four different treatment groups (*n* = 6). **f** The tumor volume in individual mouse. **g** The mean ± S.D. value from the six mice in each treatment group. **h** The Kaplan–Meier curves and log-rank test of overall survival analysis of LLC-tumor bearing C57BL/6 mice in the four different treatment groups (*n* = 6). *p < 0.05, **p < 0.01, ***p < 0.001

To determine whether upregulation of PD-L1 by MMC would affect the antitumor immune activity, H460 cells were pretreated with MMC for 24 h, and then co-cultured with PBMCs for another 24 h. Significantly more cell lysis was detected in H460 cells pretreated with MMC than the cells without MMC pretreatment (Fig. 2a). Moreover, remarkably higher level of TNF-α was detected in CD3 + T-cell population from PBMCs after incubation with MMC-pretreated H460 cells (Fig. 2b). These results suggest that T-cell activation was enhanced by MMC. A stable PD-L1 knockout PD-L1/KO H460 cell was established to verify the involvement of PD-L1 in the enhanced anticancer effect from the combination of MMC and PD-L1 blockade (Fig. 2c). T-cell-mediated tumor cell killing in PD-L1/KO H460 cells and the wild-type control H460 cells was evaluated by Giemsa staining after PD-L1 antibody treatment, with or without pretreatment by MMC. Combination of MMC and PD-L1 antibody was found to exhibit remarkably higher anticancer effect than either MMC or PD-L1 antibody alone in both wild-type H460 and PD-L1/KO H460 cells, albeit more pronounced effect was observed in the latter cells (Fig. 2d). Granzyme B is a serine protease known to mediate the apoptotic effect of cytotoxic T lymphocytes. Importantly, more granzyme B release from CD8 + T cells was observed in PBMCs after incubation with MMC-pretreated H460 cells than no-pretreatment H460 cells (Fig. 2e). Lewis lung carcinoma model was established in the flanks of C57BL/6 mice to evaluate the combination anticancer effect of MMC and PD-L1 antibody in vivo. When the tumor volumes reached ~100 mm^3^, mice were randomized to receive treatment with MMC alone, PD-L1 antibody alone, and the combination of MMC and PD-L1 antibody, or saline + isotope control IgG as intraperitoneal injection. The combination of MMC and PD-L1 antibody was shown to cause a significantly more prolonged retardation of tumor growth when compared with IgG-treated control group and the other monotherapy groups (Fig. 2f, g). Moreover, the combination of MMC and PD-L1 antibody also gave rise to the best survival among the four treatment groups (Fig. 2h).

### MMC increased MHC-I expression in tumor cells

**Fig. 3:**
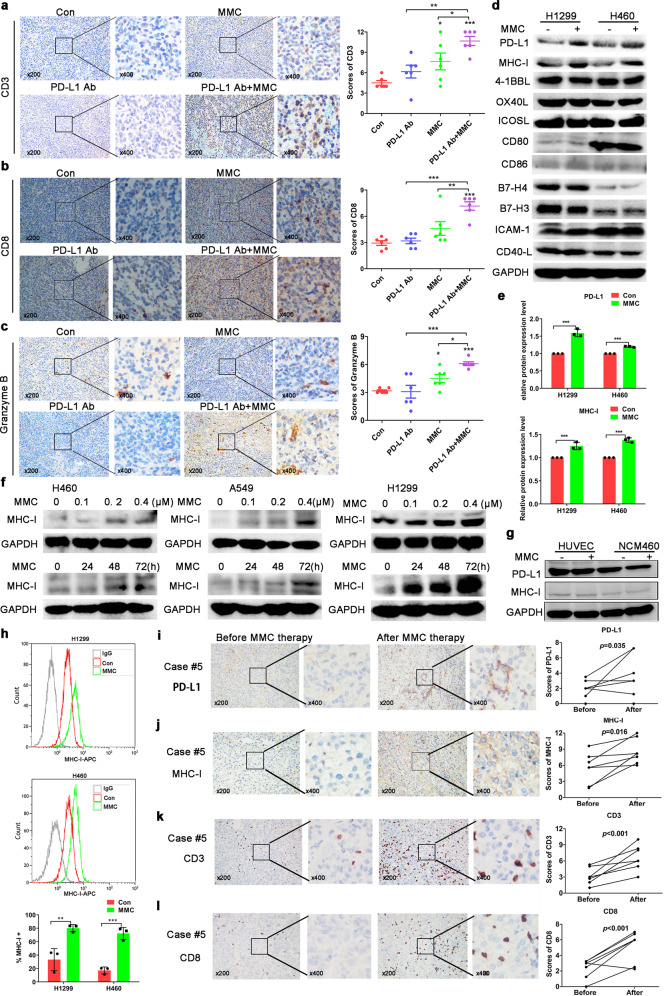
MMC increased MHC-I expression in tumor tissues. **a**–**c** Representative images showing the immunohistochemical staining of CD3, CD8, and granzyme B in tumors of LLC-tumor bearing C57BL/6 mice (four different treatment groups; *n* = 6). The staining scores were presented as means ± S.D in the scatter plot. **d**, **e** Western blot analysis of protein expression of different immune molecules in NSCLC cells following MMC treatment for 48 h. **f** Western blot analysis showing the upregulation of MHC-I protein expression in the three NSCLC cells after MMC treatment at the different indicated concentrations and time points. **g** Western blot analysis of PD-L1 and MHC-I protein expression in two normal cells following MMC treatment for 48 h. **h** Flow cytometric analysis of the cell surface expression of MHC-I. Normal isotype IgG was used as a control to show background staining. **i**–**l** Representative images from immunohistochemical staining of CD3, CD8, PD-L1, and MHC-I in tumor specimens obtained from seven patients before and after MMC therapy

To investigate the potentiation of T cells response by MMC after PD-L1 antibody treatment, the number of tumor-infiltrating T cells and their activation were determined in the tumor tissues of C57BL/6. By Immunohistochemical staining, both the number of tumor-infiltrating CD3+ and CD8 + T cells were notably increased after the combination treatment by MMC and PD-L1 antibody than the two individual monotherapies alone (Fig. 3a, b). Moreover, the T cells activation marker granzyme B was also evidently increased in the combination treatment group (Fig. 3c). To investigate the mechanisms leading the increase of TILs, several co-stimulatory or co-inhibitory molecules involved in T cells activation were detected. Tumor cells are able to escape specific immune responses mediated by cytotoxic T cells by down-regulating the surface expression of the class I major histocompatibility complex (MHC-I), which is required for recognition and the subsequent killing effect. To this end, there was an increase of MHC-I expression accompanying the upregulation of PD-L1 in NSCLC cells after incubation with MMC for 48 h (Fig. 3d, e). Indeed, MMC was also found to increase MHC-I expression in NSCLC cells in concentration- and time-dependent manners (Fig. 3f). However, we detected the expressions of PD-L1 and MHC-I after MMC treatment in two normal cells, including HUVEC (human umbilical vein endothelial cells) and NCM460 (normal-derived colon mucosa cells). Neither the PD-L1 nor MHC-I expression was affected by MMC (Fig. 3g). The expression of MHC-I on cell surface of H1299 and H460 cells was also found to be elevated by MMC (Fig. 3h). To further confirm the results, the alterations of TILs in tumor specimens from patients before and after receiving MMC therapy were evaluated. Parallel to the elevation of PD-L1 and MHC-I expression in the tumor tissues after MMC therapy (Fig. 3I, j), the amount of CD3+ and CD8+ infiltrating lymphocytes in the tumor tissues were also found to be significantly increased after MMC therapy (Fig. 3l, m).

### MMC increased MHC-I expression via upregulation of p65

**Fig. 4:**
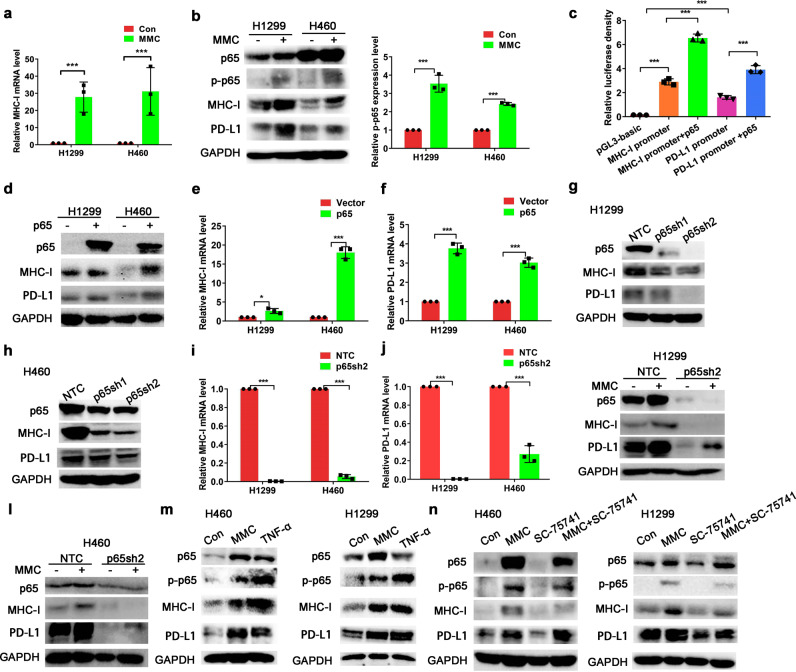
MMC increased MHC-I expression via activation of p65. **a** Quantitative real-time PCR data showing MHC-I mRNA levels in NSCLC cells after incubation with MMC for 48 h (0.4 μM). **b** Western blot analysis of total p65 and phospho-p65 in NSCLC cells after 48-h treatment with MMC (0.4 μM). **c** Luciferase activity in 293T cells transiently co-transfected with p65 or empty vector and MHC-I promoter or PD-L1 promoter constructs in the presence or absence of p65 expression vector for 48 h. **d** Western blot analysis showing the upregulation of MHC-I and PD-L1 protein expression by ectopic expression of p65 in NSCLC cells. **e**, **f** The mRNA levels of MHC-I and PD-L1 in NSCLC cells incubated with MMC for 48 h (0.4 μM). **g**, **h** Western blot analysis showing the knockdown of p65 protein by shRNA; and the MHC-I and PD-L1 expression in the p65 knockdown cells. NTC, no template control. **i**, **j** The mRNA levels of MHC-I and PD-L1 in p65 knockdown cells. **k**, **l** Western blot analysis of PD-L1 and MHC-I protein expression in p65 knockdown cells after MMC treatment (0.4 μM,48 h). **m** The PD-L1 and MHC-I protein expression in NSCLC cells treated with TNF-α (20 ng/ml) or MMC (0.4 μM) for 4 8 h. **n** PD-L1 and MHC-I protein expression in cells treated with MMC (0.4 μM) in the presence or absence of p65 inhibitor (SC-75741 (10 μM)) for 48 h. The H460 and H1299 cells were pre-treated with 10 μM SC-75741 for 2 h. *p < 0.05, **p < 0.01, ***p < 0.001

The MHC-I mRNA levels were notably elevated in NSCLC cells (H1299 and H460) after 48 h treatment with MMC (0.4 μM; Fig. 4a). The transcription factor NF-κB is an important mediator of cellular responses to inflammatory stimuli.^32,33^ To this end, NF-κB p65 has been reported to regulate MHC-I and PD-L1 expression transcriptionally.^25^ The effect of MMC on the expression of p65 sub-unit of NF-κB in NSCLC cells after 48 h treatment of MMC was evaluated. Both p65 and its phosphorylated form p-p65 were increased by MMC treatment (Fig. 4b). Data from the luciferase reporter assay showed that p65 significantly upregulated the MHC-I and PD-L1 promoter-driven luciferase activity (Fig. 4c). Moreover, both mRNA and protein levels of MHC-I and PD-L1 in NSCLC cells were elevated by p65 overexpression (Fig. 4d–f). On the other hand, the protein expressions of MHC-I and PD-L1 were also decreased by silencing of p65 (Fig. 4g–j). To investigate whether p65 is essential for MMC-induced upregulation of MHC-I and PD-L1, protein expression of MHC-I and PD-L1 was evaluated in p65-knockdown H1299 or H460 cells after MMC treatment. While MMC was able to induce p65, MHC-I, and PD-L1 expression notably in control cells, silencing of p65 was shown to attenuate MMC-induced MHC-I and PD-L1 upregulation (Fig. 4k, l). TNF-α is needed for activation of NF-kB/p65-dependent transcription. To verify whether activation of p65 was related to MMC-induced MHC-I and PD-L1 upregulation, p65 phosphorylation and expression of MHC-I and PD-L1 were detected in H460/H1299 cells after treatment with MMC or TNF-α. The phosphorylation of p65 and total p65 expression were increased, along with the upregulation of both MHC-I and PD-L1, in the presence of MMC or TNF-α (Fig. 4m). Interestingly, SC-75741 (an inhibitor that prevents the transcription activity of p65) was found to inhibit the induction of the MHC-I expression by MMC in both H1299 and H460 (Fig. 4n). In contrast, the upregulation of PD-L1 expression by MMC was not affected in the presence of SC-75741 (Fig. 4n), suggesting that MMC-induced upregulation of PD-L1 expression is independent of the transcriptional activity of p65.

### MMC promoted binding of c-JUN to the PD-L1 promoter to upregulate PD-L1 expression transcriptionally

**Fig. 5:**
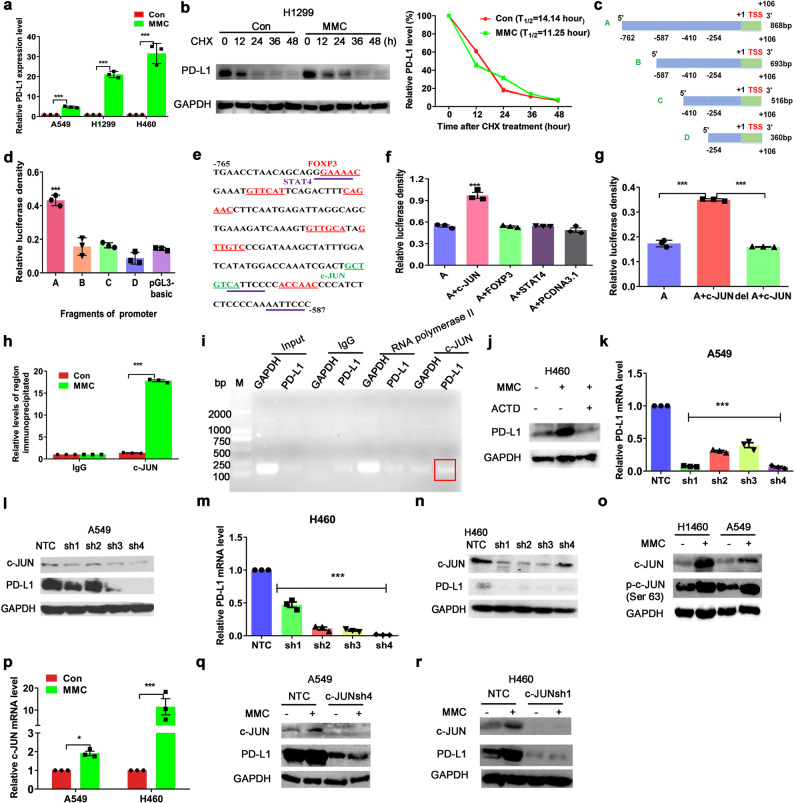
MMC increased PD-L1 expression via activation of c-JUN. **a** Quantitative real time PCR analysis of PD-L1 mRNA levels in NSCLC cells after incubation with MMC for 48 h (0.4 μM). **b** Western blot analysis of PD-L1 protein expression in H1299 cells after incubation with MMC (0.4 μM), followed by treatment of cycloheximide (CHX, 20 μM) for the indicated time points. Cell lysates were immunoblotted by the indicated antibodies (left). Data were quantified using Image J software (right). **c** Graphical representation showing the four fragments of PD-L1 promoter was cloned into pGL3-basic vector for the luciferase reporter gene assay. **d** Luciferase activity of the 4 PD-L1 promoter fragments constructs and empty luciferase vector pGL3-basic. **e** The −765 to −587-bp sequence of the 5′ -flanking region of PD-L1 promoter is shown. Underlined sequences were potential binding sites for common transcription factors as predicted by PROMO. **f** Luciferase activity of PD-L1 promoter fragment A constructs co-transfected with expression vector for c-JUN, STAT4, FOXP3, or pCDNA 3.1 vectors respectively. **g** Relative luciferase activity of c-JUN binding site-deleted fragment A. **h** c-JUN or IgG was immunoprecipitated from the nuclear lysate of 1 × 10^6^ H460 cells and ChIP was used to demonstrate the enrichment of PD-L1 promoter at the c-JUN binding site. **i** Chromatin immunoprecipitation assay analyzing the binding of c-JUN to the PD-L1 promoter. Rabbit IgG was used a negative control. The binding of RNA polymerase II to the constitutive GAPDH promoter was used as a positive control. **j** The PD-L1 expression in H460 cells treated with MMC (0.4 μM) followed by actinomycin D treatment (1 mg/ml) for 5 h. **k**–**n** The mRNA and protein levels of PD-L1 in c-JUN knockdown cells. NTC no template control. **o**, **p** The c-JUN and its mRNA levels in cells after MMC treatment (0.4 μM, 48 h). **q**, **r** Western blot analysis of PD-L1 expression in c-JUN knockdown cells with MMC treatment (0.4 μM, 48 h). All conditions were evaluated in triplicates. *p < 0.05, **p < 0.01, ***p < 0.001

In all NSCLC cells (A549, H1299, and H460) tested, the PD-L1 mRNA levels were significantly increased after MMC treatment for 48 h (Fig. 5a). PD-L1 protein stability was also detected in H1299 cells, which was not affected by MMC treatment (Fig. 5b). In order to investigate the transcriptional regulation of PD-L1 by MMC, luciferase reporter gene assay was carried out in 293T cells transiently transfected with pGL3-basic luciferase constructs containing four different PD-L1 promoter regions with progressive 5′ deletion (Fig. 5c). The longest PD-L1 promoter sequence (p868) was shown to exhibit the highest luciferase activity among all four promoter regions tested (Fig. 5d). This finding suggests that a core element within the PD-L1 promoter region (−762 to −587 bp) is crucial to drive transcription and the observed luciferase activity. According to PROMO (http://alggen.lsi.upc.es/) and JASPAR database (http://jaspar.genereg.net/), a few candidate transcription factors (including c-JUN, STAT4, and FOXP3 (Fig. 5e)) were predicted to bind with the core PD-L1 promoter element to drive the gene transcription. To further confirm the identity of the transcription factor(s) which binds to the PD-L1 core promoter and drives its transcription, expression vectors (pCDNA3.1/c-JUN, STAT4, or FOXP3) were transfected to 293T cells and the luciferase reporter gene assay repeated using the luciferase construct harboring the core PD-L1 promoter region (−762 to −587 bp). Only pCDNA3.1/c-JUN was found to activate the PD-L1 promoter-driven luciferase activity (Fig. 5f). Moreover and importantly, when the c-JUN binding sites were deleted from the PD-L1 promoter core promoter region (DeltA), the PD-L1 promoter-driven luciferase activity dropped back to the baseline level (Fig. 5g). By ChIP assay performed in H460 cells, immunoprecipitation of the PD-L1 chromatin using antibody against c-JUN revealed the substantial increase in the association of c-JUN with the core PD-L1 promoter region after MMC treatment (Fig. 5h). Immunoprecipitation using the isotope control rabbit IgG was used as a negative control. To demonstrate the specificity of the ChIP assay, the binding of c-JUN and RNA polymerase II to the constitutive GAPDH promoter versus the core PD-L1 promoter was also analyzed (Fig. 5i). No PCR signal was obtained from immunoprecipitated samples when normal IgG was used. While the association of RNA polymerase II with both GAPDH and PD-L1 promoter was detected using the RNA polymerase II-pulled down chromatin, c-JUN was shown to specifically bind with the core PD-L1 promoter in the immunoprecipitated chromatin using c-JUN antibody (Fig. 5i). The transcription inhibitor actinomycin D was shown to block MMC-mediated PD-L1 upregulation (Fig. 5j). Therefore, it is likely that c-JUN bind to the PD-L1 gene promoter to transcriptionally upregulate PD-L1 expression. To confirm the role of c-JUN as a transcription factor regulating the basal PD-L1 expression in NSCLC cells, c-JUN was knocked down using shRNAs. Both mRNA and protein levels of PD-L1 were significantly decreased in c-JUN-knockdown tumor cells (Fig. 5k–n). To further investigate the mechanism played by c-JUN in MMC-mediated upregulation of PD-L1 expression, c-JUN expression was detected after MMC treatment for 48 h in A549 and H460 cells. Both mRNA and protein levels of c-JUN were found to be remarkably increased by MMC treatment (Fig. 5o, p). The activated form phospho-c-JUN was also found to be significantly increased after MMC treatment (Fig. 5o). Importantly, in c-JUN-knockdown NSCLC cells (A549 and H460), the upregulation of PD-L1 by MMC was abrogated (Fig. 5q, r).

### Upregulation of PD-L1 expression by MMC depended on the co-operation of STAT3

**Fig. 6:**
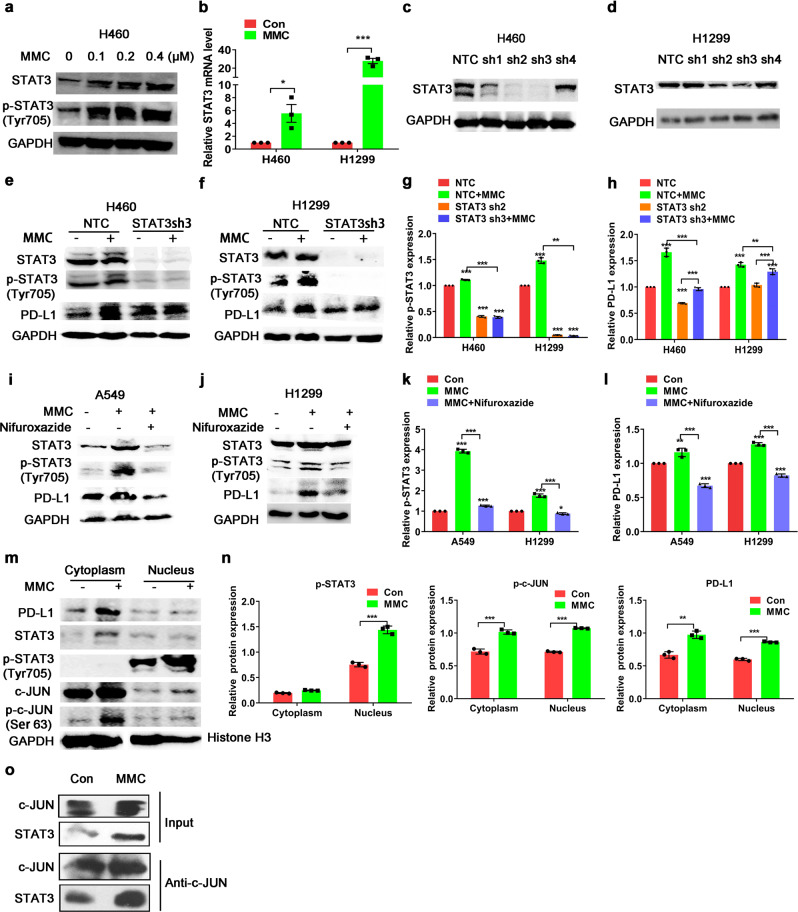
C-JUN cooperated with its co-factor STAT3 to upregulate PD-L1. **a** Western blot analysis showing total STAT3 and phospho-STAT3 protein expression in H460 cells after MMC treatment (0–0.4 μM, 48 h). **b** Quantitative real-time PCR analysis of STAT3 mRNA levels in MMC treated cells (0.4 μM, 48 h). Results are represented as means ± S.D. from triplicate experiments. **c**, **d** Western blot analysis showing the knockdown of total STAT3 by four different shRNAs (sh1-sh4) in H460 and H1299 cells. NTC no template control. **e**–**h** Western blot analysis showing PD-L1 protein expression in STAT3 knockdown cells with or without MMC treatment. Expression levels of PD-L1 or STAT3 protein was normalized with GAPDH and data are presented as relative ratio to the untreated cells (**g**, **h**). **i**–**l** PD-L1 protein expression in cells after treated with MMC in the presence or absence of nifuroxazide (a STAT3 inhibitor). A549 and H1299 cells were pretreated with 10 μM Nifuroxazide for 5 h. Expression level of PD-L1 or STAT3 protein was normalized with GAPDH and data are presented as relative ratio to the untreated cells (**k**, **l**). **m**, **n** The cytoplasm and nuclear translocation of c-JUN and STAT3 analyzed using cell fractionations in H460 cells after MMC treatment (0.4 μM, 48 h). The expression level of the protein was normalized with GAPDH or Histone H3. **o** The co-immunoprecipitation analysis of interaction of endogenous c-JUN and STAT3 proteins in H460 cells treated with MMC (0.4 μM, 48 h). *p < 0.05, **p < 0.01, ***p < 0.001

Previous studies reported that c-JUN interacted with STAT3 to form a complex to regulate the expression of their target genes.^34^ To examine whether STAT3 contributed to MMC-mediated PD-L1 upregulation, the STAT3 expression was detected in H1299 and H460 cells after MMC treatment. Both the mRNA and protein levels of STAT3 in the NSCLC cells were increased by MMC (Fig. 6a, b). Importantly, the increase of PD-L1 expression by MMC was abrogated in STAT3-knockdown cells (Fig. 6c–h). Consistent with this finding, MMC failed to upregulate PD-L1 expression in the NSCLC cells in the presence of nifuroxazide (an inhibitor preventing the activation of STAT3; Fig. 6i–l). As p-c-JUN and p-STAT3 were translocated to the nucleus in order to exert their transcription activity, nuclear and cytoplasmic fractions from H460 cells with or without MMC treatment were isolated and detected. Accompanied with the elevated cytoplasmic expression of PD-L1 after MMC treatment, the cytoplasmic and nuclear c-JUN and p-c-JUN expression were also found to be elevated by MMC (Fig. 6m, n). On the other hand, the transcriptionally active form of STAT3 (p-STAT3) was also upregulated in the nucleus (Fig. 6m, n). Moreover, the interaction between c-JUN and STAT3 was enhanced in H460 cells after MMC treatment (Fig. 6o). Thus, MMC was shown to modulate the expression and interaction of c-JUN and STAT3 to upregulate PD-L1 expression in vitro.

### MMC stimulated MHC-I and PD-L1 expressions by activating ERK

**Fig. 7:**
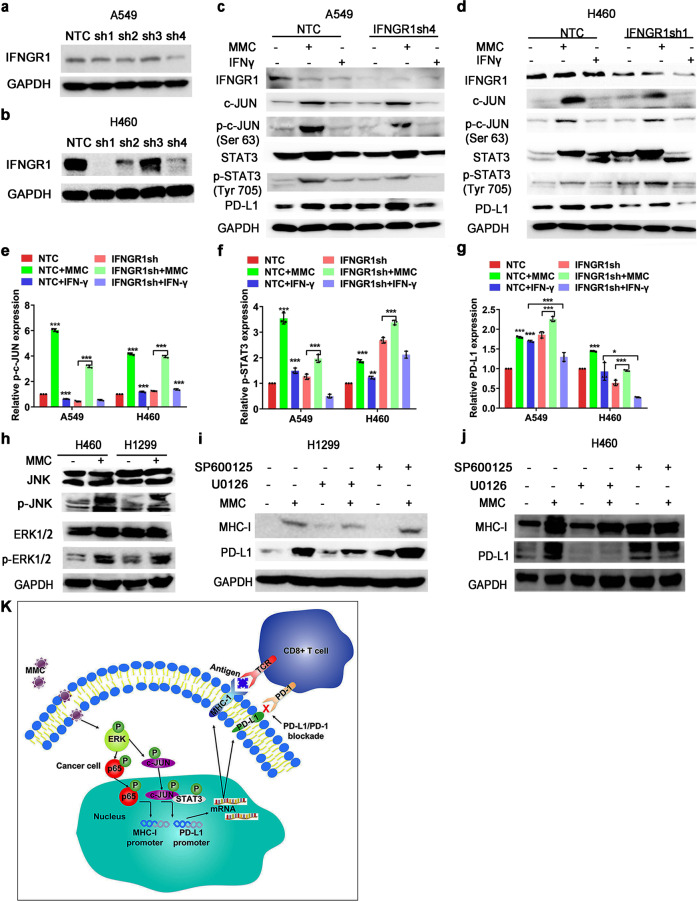
MMC induced MHC-I and PD-L1 expression via ERK signaling. **a**, **b** Western blot analysis showing the knockdown of IFNGR1 by four different shRNAs (sh1-sh4) in A549 and H460 cells. **c**–**g** Western blot analysis showing the PD-L1 protein expression in IFNGR1-knockdown cells with or without MMC (0.4 μM, 48 h) or IFN-γ (1 ng/mL) treatment. The IFN-γ (1 ng/ml) was used a positive control. The expression level of the protein was normalized with GAPDH. Data are presented as relative ratio to the untreated cells (**e**–**g**). *p < 0.05; **p < 0.01; ***p < 0.001. **h** Western blot analysis showing ERK and JNK protein expressions in MMC treated cells (0.4 μM, 48 h). **i**, **j** The PD-L1 and MHC-I expression in MMC treated cells (0.4 μM, 48 h) in the presence of an ERK inhibitor (U0126 (20 μM)) or a JNK inhibitor (SP600125 (15 μM)). The cells were pretreated with U0126 or SP600125 for 3 h. The expression level of the protein was normalized with GAPDH. *p < 0.05, **p < 0.01, ***p < 0.001. **k** A schematic diagram summarizing the molecular mechanisms underlying the upregulation pf PD-L1 and MHC-I by MMC in NCSLC cells. MMC activates the ERK pathway, which subsequently enhances the binding of c-JUN to the PD-L1 promoter and recruits its co-factor STAT3 to increase PD-L1 expression. The upregulated ERK pathway also activates p65 to increase the MHC-I expression. The up-regulations of PD-L1 and MHC-I then induce more and more CD3+ and CD8+ T-cell infiltration in tumor tissue and kill the tumor cells

PD-L1 is known to be regulated by two major mechanisms, namely IFN-γ dependent and independent ways. In order to verify whether IFN-γ signaling is involved in the upregulation of PD-L1 expression by MMC, A549, and H460 cells transfected with control shRNA or IFNGR1 shRNAs (Fig. 7a, b) were incubated with or without MMC treatment. Both in control and IFNGR1-knockdown cells (A549 and H460), expression of PD-L1, c-JUN, STAT3, and their phosphonate forms were increased after MMC treatment (Fig. 7c–g). On the other hand, IFN-γ was found to significantly increase PD-L1 expression, but this effect was abrogated in IFNGR1-knockdown cells (Fig. 7c–g). Therefore, the increase of PD-L1 following MMC treatment was mediated by the c-JUN/STAT3 signaling pathway rather than via an IFN-γ-dependent manner. MMC has been reported to increase the activity of c-JUN via ERK and JNK signaling.^35^ Moreover, p65 is a downstream transcription factor activated by the MAPK pathway.^36^ Therefore, the effect of MMC on p-ERK1/2 and p-JNK protein levels was detected. The phosphorylation of ERK and JNK were both elevated by MMC in both H1299 and H460 cells (Fig. 7h). To further delineate the involvement of ERK and JNK signaling, the effects of a MEK inhibitor (U0126) or a JNK inhibitor (SP600125) on MMC-induced upregulation of PD-L1 and MHC-I expression were investigated. While the induction of PD-L1 and MHC-I by MMC was not affected by SP600125, U0126 was shown to significantly reduce the upregulation of the PD-L1 and MHC-I levels by MMC (Fig. 7i, j). Thus, these findings indicated that activation of the ERK pathway plays a critical role in the upregulation of MHC-I and PD-L1 by MMC in NSCLC cells. A graphical scheme summarizing the mechanisms contributing to the synergistic combination of PD-L1 antibody and MMC in NSCLC cells is shown in Fig. 7k.

## Discussion

The PD-1/PD-L1 checkpoint inhibitors are revolutionizing cancer therapy, which induce durable anti-tumor responses and overall survival benefit in a wide variety of cancer types. However, the low response rate of cancer cells remains a big challenge. There is growing evidence that cytotoxic chemotherapy alters the immunosuppressive tumor microenvironment and activates immune response to kill tumor cells.^11^ Thus, in this study, a classical cytotoxic chemotherapeutic drug MMC was shown to upregulate PD-L1 and MHC-I expressions and enhanced the antitumor immune response in NSCLC cells. Pretreatment with MMC increased the cell lysis mediated by lymphocytes and promoted secretion of granzyme B and TNF-α from CD8+ T cells, which often correlated with enhanced anti-tumor cytotoxicity.^37^ Our data also demonstrate that MMC synergized with PD-L1 blocking antibodies in vivo. Compared with either PD-L1 antibody or MMC monotherapy alone, the combination therapy was shown to be more effective in extending overall survival and inhibiting tumour growth in tumor-bearing C57BL/6 mice compared to monotherpies. To study the underlying mechanisms contributing to the synergistic effect of MMC and PD-L1 antibody, the amount and activity of TILs were investigated in the mice models. The abundances of both the CD3+ and CD8+ TILs at the tumor sites were found to be increased after MMC treatment. Besides increasing the number of TILs, the MMC + PD-L1 antibody combination was also shown to increase the release of granzyme B from the TILs. A few chemotherapeutic drugs have been reported to alter the tumor microenvironment to activate immune response,^11,38^ and increase the number of cytotoxic CD8^+^T cells within the tumor.^39–41^ Therefore, the key immune molecules involved in T cells activation, including co-stimulatory and co-inhibitory molecules were evaluated. Accompanied with the upregulation of PD-L1 in MMC treated cells, the MHC-I molecule was also increased, presumably promoting the recognition of tumor cells by the acquired immune system. In contrast, other co-stimulatory and co-inhibitory molecules were not affected by MMC treatment. To this end, high surface expression of MHC-I molecules on cancer cells has been reported to elevate the amount of tumor-reactive CD8+ T-cell to elicit the antitumor immune response.^42^ Therefore, the combination of PD-L1 antibody and MMC may induce tumor regression by attenuating the immunosuppressive PD-1/PD-L1 signal to achieve maximal immune activation. Importantly, in tumor specimens obtained from patients treated with MMC, an increase of PD-L1 and MHC-I expression was also observed. Moreover, the abundances of CD3+ and CD8+ TILs at the tumor tissues were also found to be remarkably increased in patients’ specimens. Consistent with previous studies,^43,44^ our findings also confirmed that MMC could serve as an adjuvant for antitumor immunity. There is emerging evidence to support that several chemotherapeutic agents may function as a vaccine and provide antitumor immune response.^45^ These chemotherapeutic drugs increased the leakage of tumor antigens to antigen-presenting cells, thereby resulting in increased antitumor responses.^45^ Moreover, neither the PD-L1 nor MHC-I expression was affected by MMC treatment in normal cells, which suggest that MMC does not increase the toxicity of immunotherapy in normal cells. Our data clearly demonstrate that MMC could induce an immune-reactivation status through accumulation of CD8+ T cells by upregulation of MHC-I expression.

Activation of the NF-κB transcription factor is associated with tumor progression and inflammation responses.^46–49^ It has been demonstrated that activation of NF-κB signaling was related to the high MHC-I expression in neuroblastoma previously.^50^ In our study, the p65 sub-unit of NF-κB was also shown to induce overexpression of MHC-I and PD-L1 in NSCLC cells in our study, but specific p65 inhibitor failed to suppress MMC-induced PD-L1 upregulation. MMC was found to upregulate PD-L1 expression transcriptionally in NSCLC cells because it could be reversed by the transcription inhibitor actinomycin D. A key *cis*-element within the PD-L1 promoter essential for the MMC-mediated induction of PD-L1 was subsequently identified.

This study also evaluated the detailed molecular mechanism leading to the induction of PD-L1 by MMC treatment. C-JUN was identified as a unique key transcription factor that binds to the PD-L1 promoter to promote the gene transcription. The activity of c-JUN in NSCLC cells is closely related to the levels of PD-L1 expression. Knock-downing of c-JUN is necessary and sufficient to suppress the basal expression of PD-L1 in NSCLC cells. Importantly, MMC-mediated PD-L1 upregulation was abrogated in c-JUN-knockdown NSCLC cells. STAT3 was also involved in the increase of PD-L1 expression after MMC treatment. MMC increased the expression of activated STAT3 in the nucleus and there was increased binding of c-JUN and STAT3 upon MMC treatment to promote PD-L1 transcription. The c-JUN/STAT3/PD-L1 axis is an important pathway in determining the immune evasion potential of cancer cells. In fact, our observations are consistent with a recent report that constitutive c-JUN activity induced PD-L1 expression in Hodgkin lymphoma.^35^ The enhancer/promoter elements of inducible immune regulatory ligands may play a critical role in cancer immune evasion.

The regulation of PD-L1 is known to be mediated by two classical pathways, IFN-γ dependent way,^51^ and IFN-γ independent way (including MAPK,^52^ phosphoinositide 3-kinase/protein kinase B (PI3K/PKB), and the Janus kinase/signal transducers and activators of transcription (JAK/STATs^53^). Our data showed that MMC-induced PD-L1 upregulation was not IFN-γ dependent. It has been reported that PD-L1 expression was regulated by MEK activation in glioma or myeloma.^51^ The importance of MEK/ERK pathway in regulating apoptosis after MMC treatment has also been previously suggested.^54^ ERK1/2 and JNK are direct kinases modulating c-JUN activity,^35^ therefore these kinases might be involved in the modulation of PD-L1 expression in response to MMC. Moreover, p65 is also known to be a downstream transcription factor in the MAPK pathway.^36^ In MMC-treated NSCLC cells, both the ERK and JNK were found to be highly activated which accompanied with the upregulation of PD-L1 expression. Interestingly, the inhibition of ERK activity by a MEK inhibitor (U0126), was able to suppress the upregulation of PD-L1 and MHC-I expression in MMC-treated NSCLC cells. To this end, MEK inhibition has also been shown to reverse PD-L1–mediated inhibition of cytotoxic T-cell function in the immunotherapy of acute myeloid leukemia cells.^55^ Thus, activated ERK signaling is important in allowing the upregulation of PD-L1 and MHC-I by MMC, presumably contributing to the synergistic combination of PD-L1 antibody and MMC.

## Conclusion

In this study, MMC was found to upregulate PD-L1 and MHC-I expression in NSCLC and enhanced the efficacy of PD-L1 blockade in vitro and in vivo, which was associated with the increase of TILs in tumor mass to mediate the antitumor immunity. The upregulation of PD-L1 expression was mediated via ERK signaling through the activation of c-JUN and increased interaction between c-JUN and STAT3. On the other hand, MHC-I was increased by the activation of p65. Taken together, this study advocates the novel combination of PD-L1 antibody and MMC to increase the response rate of PD-1/PD-L1 based immunotherapy in treating NSCLC.

## Supplementary information

Supplementary materials

Supplementary figure 1

## Data Availability

All data generated or analyzed during this study are included in this published article. All the original data would be deposited in the database (http://www.researchdata.org.cn/). The approved RDD number is RDDB2020000827.
