# Supplementary material for: Mitomycin C enhanced the efficacy of PD-L1 blockade in non-small cell lung cancer
Source: Signal Transduct Target Ther. 2020 Aug 28;5:141. doi: 10.1038/s41392-020-0200-4 (PMC7452895; doi:10.1038/s41392-020-0200-4)
Supplement: Supplementary file 1 — Supplementary materials [file 41392_2020_200_MOESM1_ESM.docx]

**Supplementary materials**

**Supplementary figure 1. The MTT assay of MMC.**

(a-i) The cell vialibity of different cancer cells in different concentrations of MMC.
