# Supplementary figures and images for: Mitomycin C enhanced the efficacy of PD-L1 blockade in non-small cell lung cancer
Source: Signal Transduct Target Ther. 2020 Aug 28;5:141. doi: 10.1038/s41392-020-0200-4 (PMC7452895; doi:10.1038/s41392-020-0200-4)

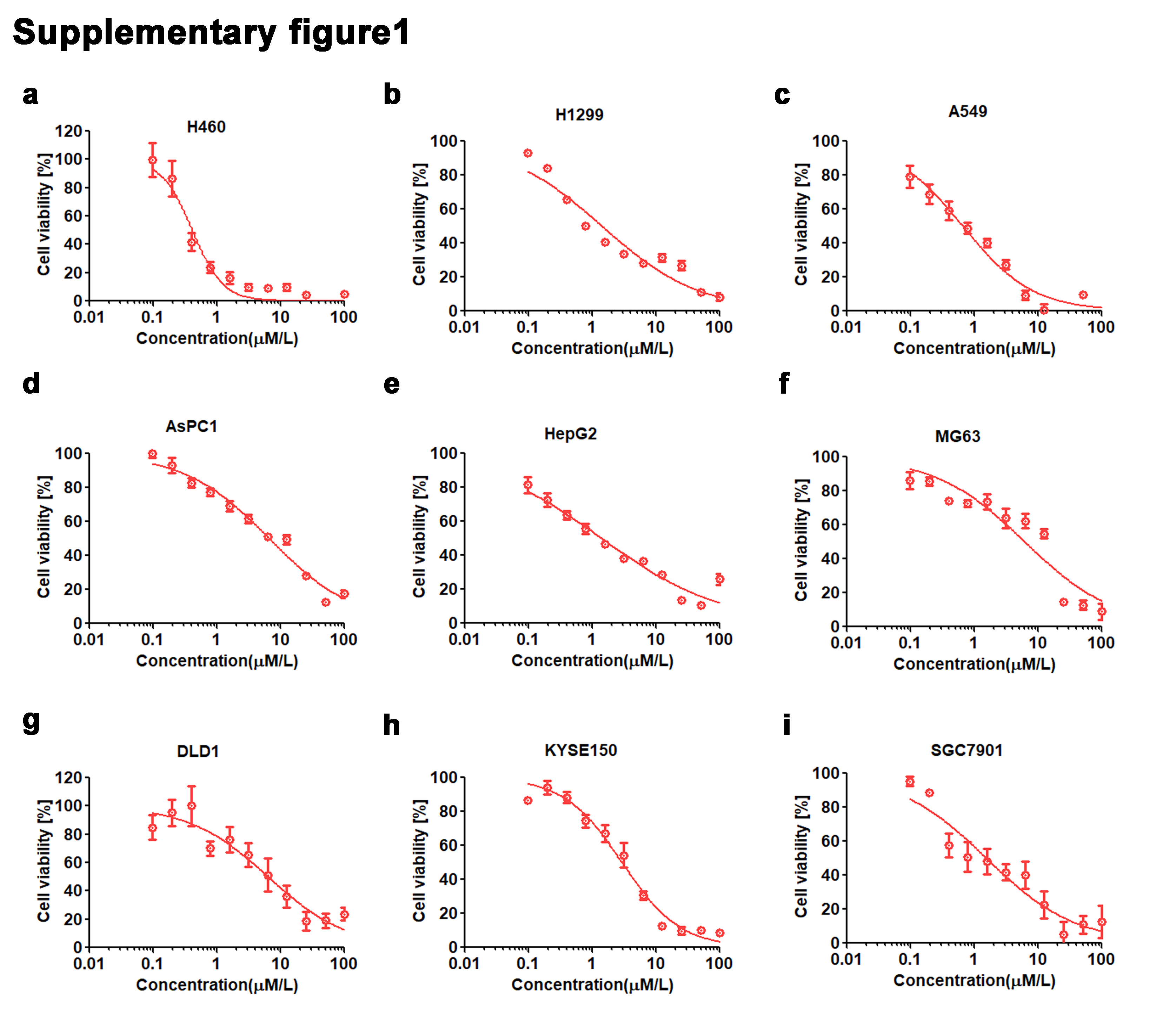

Supplement: Supplementary file 2 — Supplementary figure 1 [file 41392_2020_200_MOESM2_ESM.jpg]
